# Sequencing CTLA-4 blockade with cell-based immunotherapy for prostate cancer

**DOI:** 10.1186/1479-5876-11-89

**Published:** 2013-04-04

**Authors:** Satoshi Wada, Christopher M Jackson, Kiyoshi Yoshimura, Hung-Rong Yen, Derese Getnet, Timothy J Harris, Monica V Goldberg, Tullia C Bruno, Joseph F Grosso, Nicholas Durham, George J Netto, Drew M Pardoll, Charles G Drake

**Affiliations:** 1 Department of Oncology, Johns Hopkins University, School of Medicine, Baltimore, Maryland 21231, USA; 2 Department of Urology, James Buchanan Brady Urological Institute, Johns Hopkins University, School of Medicine, Baltimore, Maryland 21231, USA; 3 Department of Pathology, Johns Hopkins Sidney Kimmel Comprehensive Cancer Center, Johns Hopkins University, School of Medicine, Baltimore, Maryland 21231, USA; 4 Current address: Saitama Red Cross Hospital, Saitama 338-8553, Japan; 5 Current address: Department of Digestive Surgery & Surgical Oncology, Yamaguchi University, Yamaguchi University Graduate School of Medicine, Yamaguchi 755-8505, Japan; 6 Current address: Bristol-Myers Squibb, Princeton, NJ, 08543, USA; 7 Departments of Oncology, Immunology, and Urology, Johns Hopkins Sidney Kimmel Comprehensive Cancer Center, 1650 Orleans Street, CRB 410, Baltimore, MD 21231, USA

**Keywords:** Prostate cancer, Immunotherapy, Treg, Lymphocyte, CTLA-4

## Abstract

**Background:**

The FDA recently approved an anti-CTLA-4 antibody (Iplimumab) for the treatment of metastatic melanoma. This decision was based on Phase III results, which demonstrate that blocking this immune checkpoint provides a survival advantage in patients with advanced disease. As a single agent, ipilimumab is also being clinically evaluated in advanced (metastatic, castrate-resistant) prostate cancer and two randomized, placebo-controlled Phase III studies have recently completed accrual.

**Methods:**

We used a well-described genetically engineered mouse (GEM), autochronous prostate cancer model (Pro-TRAMP) to explore the relative sequencing and dosing of anti-CTLA-4 antibody when combined with a cell-based, GM-CSF-secreting vaccine (GVAX).

**Results:**

Our results show that combined treatment results in a dramatic increase in effector CD8 T cells in the prostate gland, and enhanced tumor-antigen directed lytic function. These effects are maximized when CTLA-4 blockade is applied after, but not before, vaccination. Additional experiments, using models of metastatic disease, show that incorporation of low-dose cyclophosphamide into this combined treatment regimen results in an additional pre-clinical benefit.

**Conclusions:**

Together these studies define a combination regimen using anti-CTLA-4/GVAX immunotherapy and low-dose chemotherapy for potential translation to a clinical trial setting.

## Background

Prostate cancer is the most prevalent non-dermatologic malignancy and the third most common cause of cancer-related death among men in developed countries. In the United States, 217,730 men were diagnosed with prostate cancer in 2010 and 32,050 died from the disease [1]. While localized tumors can be cured by surgical resection or radiotherapy, and early metastatic disease is frequently responsive to androgen ablation, docetaxel-based chemotherapy is a mainstay of treatment in the setting of progression from androgen-sensitive to castrate-resistant disease [2]. Unfortunately, the majority of these men derive only modest benefit from chemotherapy [3]. Recently, metastatic castrate-resistant prostate cancer (mCRPC) has been shown to remain sensitive to hormonal manipulation [4], and the androgen biosynthesis inhibitor abiraterone acetate is now FDA-approved in the post-docetaxel setting [5]. The novel anti-androgen enzalutamide (MDV3100) has also completed Phase III testing, with positive data reported [6]. Despite these important advances, mCRPC remains incurable in the majority of men, necessitating development of alternative treatment modalities.

With the landmark FDA approval of sipleucel-T (Provenge), prostate cancer emerged as a valid target for cancer immunotherapy [7]. Prostate cancer may be especially well-suited to this treatment approach given the non-vital nature of the prostate gland and the existence of a number of well-characterized prostate-restricted antigens [8]. In addition to sipleucel-T, several other immunotherapies for advanced prostate cancer have progressed to Phase II or Phase III clinical trials. One such therapy is GVAX (GM-CSF immunotherapy for cancer), which involves intradermal administration of irradiated, allogeneic tumor cells genetically modified to secrete granulocyte-macrophage colony-stimulating factor (GM-CSF) [9]. Despite promising results in preclinical and early clinical testing, two Phase III clinical trials of GVAX for advanced castration-resistant prostate cancer were halted [8]. There are a variety of potential reasons for the failure of GVAX immunotherapy to demonstrate clinical benefit in prostate cancer [8]. One possibility is that repeated exposure to high doses of GM-CSF could drive the induction of a regulatory population of cells known as myeloid-derived suppressor cells [10]. A second, more intriguing possibility is that persistent exposure to tumor antigens in the setting of advanced disease induces T cell tolerance, thus limiting the effectiveness of a vaccine strategy. Emerging data suggest that this effect may be at least partially obviated by combining immunotherapy (i.e. vaccines) with agents designed to block the immune checkpoints that limit an anti-tumor immune response [11].

Ipilimumab (Yervoy, BMS Princeton, NJ), a fully human anti-Cytotoxic T Lymphocyte Antigen-4 (CTLA-4) monoclonal antibody, was recently approved by the FDA for the treatment of metastatic and unresectable melanoma [12]. CTLA-4 is the most extensively studied immune checkpoint molecule, and is expressed on activated T effector cells (Teffs) [13] and T regulatory cells (Tregs) [14]. In contrast to CD28/B7 binding, which acts as a co-stimulatory signal to promote T cell activation and proliferation, the binding of CTLA-4 to B7 transmits an inhibitory signal. While CTLA-4 blockade has been shown to have powerful anti-cancer effects in some patients, it has also been associated with a significant risk of autoimmune toxicity. For example, a Phase III clinical trial of ipilimumab in patients with metastatic melanoma reported a survival benefit and an overall response rate of 11% with ipilimumab alone; however, approximately 60% of patients demonstrated immune-related adverse events and 7 patients (1.1%) died from immune-related adverse events (IRAEs) [15]. These complications may be partially reflected in mouse models as CTLA-4 knockout mice die of multi-organ failure at ~3-4 weeks of age secondary to lymphoproliferation [16].

Previous studies in murine prostate cancer models have demonstrated that CTLA-4 blockade promotes anti-tumor activity [17] and works synergistically with GM-CSF-expressing tumor vaccines [18]. Given the significant rate of IRAEs observed in the recent clinical trials described above, we sought to determine the minimum dose of anti-CTLA-4 required for additive efficacy, as well as the optimal sequence of cell-based immunotherapy (GVAX) and checkpoint blockade. These studies utilized our well-established genetically engineered mouse (GEM) model, in which the model antigen hemagglutinin (HA) is expressed in a prostate-restricted manner on the transgenic adenocarcinoma of the mouse prostate (TRAMP) background [19-23].

We subsequently tested this regimen in models of lung and liver metastasis in wild type mice as well as mice harboring synchronous prostate tumors. Finally, we added low-dose cyclophosphamide to evaluate a potential role for T regulatory cells in limiting the anti-tumor response [23]. Overall, our data confirm the finding that combining GVAX immunotherapy with CTLA-4 blockade is more effective than either treatment alone. Interestingly, we found that CTLA-4 blockade must be administered subsequent to vaccination to produce additive immunologic effects. Low-dose cyclophosphamide provided a benefit when added to this combination regimen, suggesting that CTLA-4 blockade does not completely mitigate Treg function.

## Materials and methods

All studies were completed under an protocol approved by the Johns Hopkins Animal Care and Use Committee.

### Mice

Non-transgenic B10.D2 (H-2^d^) mice were purchased from the Jackson Laboratory. Pro-HA transgenic mice express a secreted form of Hemagglutinin (HA) under control of the prostate epithelial-specific Probasin promoter [19], and are on the B10.D2 (H-2^d^) genetic background. Double transgenic (ProHA × TRAMP) mice were generated by backcrossing TRAMP (Transgenic Adenocarcinoma of the Mouse Prostate) animals on a C57/Bl6 background onto the ProHA transgenic background > 12 generations, and are homozygous for H-2^d^ at the MHC locus. As previously described [19], ProHA × TRAMP mice develop autochthonous prostate tumors that express HA as a tissue/tumor-restricted antigen. Otherwise, disease development is indistinguishable from their TRAMP counterparts [19-21]. Clone 4 transgenic mice express a CD8 TCR which is specific for the K^d^-restricted (MHC Class I) HA peptide (^542^IYSTVASSL^550^) [24]. 6.5 mice are CD4 TCR transgenic animals with a TCR specific for the I-E^d^ restricted (MHC Class II) HA peptide (^110^SFERFEIFPKE^120^) [25]. For these studies, Clone 4 and 6.5 mice were backcrossed over 12 generations onto a Thy1.1 congenic B10.D2 background. Mouse care and experimental procedures were carried out in accordance with the Institutional Animal Care and Use Committee of Johns Hopkins University under an approved protocol.

### Cell lines

The prostate adenocarcinoma cell line, TRAMP-C2, was purchased from ATCC (Manassas, VA). To construct a HA-targeted immunotherapy, TRAMP-C2 cells were transfected with full-length HA as previously described [26]. Transfectants were cloned by limiting dilution in 96 well plates, and HA expression was confirmed by staining with the HA-specific mAb H18L10-5R1 [27], which was graciously provided by Dr. J. Yewdell (NIAID). A single clone expressing high-levels of HA was selected, expanded and used in further studies. B78H1-GM is a GM-CSF secreting cell line utilized in bystander immunotherapy regimens [28], as previously described [29]. B78H1-GM cells secrete approximately 2500 ng of GM-CSF per 10^6^ cells over a 24-hour period, as determined by ELISA. The SP1 cell line was established from ProHA × TRAMP mice (prostate tumor) in our laboratory and maintained in RPMI 1640 supplemented with 10% heat inactivated FCS (HyClone, South Logan, UT), 1 mmol/L sodium pyruvate, 2 mmol/L L-glutamine, nonessential amino acids (1% of 100× stock), 25 mmol/L HEPES buffer, and 50 μmol/L 2-mercaptoethanol (C-Media).

### Reagents

Cyclophosphamide was purchased from Bristol-Myers Squibb, and diluted in PBS for intraperitoneal injection. Anti-CTLA-4 mAb was purified by protein G chromatography from supernatants of the clone UC10-4 F10-11 hybridoma obtained from American Type Culture Collection.

### Adoptive T cell transfer

Adoptive T cell transfer was performed as previously described [19]. Donor TCR transgenic mice were euthanized via CO_2_ asphyxiation. Spleens and lymph nodes were collected, homogenized, and red blood cells were lysed. CD8 or CD4 T cells were purified using Miltenyi beads according to the manufacturer’s protocol. For some experiments, purified cells were labeled for 8 minutes with CFSE (Invitrogen) by adding 0.5 μl of 5 mM stock per 1 ml cells After labeling, cells were washed twice and resuspended in HBSS. 2.5 × 10^6^ cells in 200 μL were injected per mouse by tail vein injection.

### GVAX Immunotherapy

To model allogeneic prostate GVAX immunotherapy using bystander cells, 1×10^6^ TRAMP-C2HA cells were admixed with 5×10^4^ B78H1-GM cells and irradiated (50 Gy). After three washes in HBSS, cells were resuspended in a total of 200 μl of HBSS and administered by subcutaneous injection of 100 μl into each hind limb [23]. For the metastatic treatment studies, the cell line SP1 (see below) was used to generate the GVAX vaccine, which was produced and administered in the same manner.

### Flow cytometry

Prostate glands, prostate-draining lymph nodes and spleens were harvested on predetermined days and single cell suspensions were prepared. All staining reagents were purchased from BD Pharmingen (San Diego, CA), with the exception of FoxP3, which was analyzed using an eBioscience antibody according to the manufacturer’s instructions (eBioscience, San Diego, CA). After a 30-minute incubation period, samples were washed once in PBS-1%FBS solution and analyzed using a FACScalibur instrument (BD, San Jose, CA). Intracellular cytokine analysis was performed as previously described [21]. Data were analyzed using the FlowJo software package (Treestar, Ashland, OR).

### *In vivo* CTL assay

*In vivo* CTL assays were performed as previously described [30]. Splenocytes from naive B10.D2 mice were labeled with 2.5 or 0.25 μM CFSE (Molecular Probes, Eugene, OR). 2.5 μM CFSE-labeled cells were loaded with HA class I peptide (10 μmol/L), while 0.25 μM CFSE-labeled cells were used as a negative control. Target cells were transferred intravenously (7.5 × 10^6^ cells of each population) into indicated groups of mice. Eighteen hours later, lymphocytes were isolated from the spleen and FACS analysis was performed. Histogram plots were used to determine the percentage of each target population based on the intensity of CFSE staining. Percentage-specific killing was calculated as previously described [31].

### Efficacy studies

Treatment was initiated when ProHA × TRAMP mice were 8–10 weeks of age [32]. Immunization was performed a total of three times at 1 week intervals unless otherwise indicated. Mice were euthanized at 18–20 weeks of age and the male urogenital tracts were micro-dissected under a stereomicroscope and weighed. Ventral prostate lobes were removed and fixed in 10% neutral buffered formalin followed by 70% EtOH. Tissues were then embedded in paraffin, cut into four micron sections using a cryostat, and placed onto poly-lysine-coated slides before being stained with H&E. Tumor tissues were graded in a blinded manner by two individual pathologists as previously described [21]: 0 = normal epithelium; 1 = prostatic intraepithelial neoplasia (PIN) with tufting of the epithelium but without cribiform structures; 2 = advanced PIN with cribiform structures; 3 = loss of intraductal spaces and/or basement membrane invasion (well differentiated carcinoma); 4 = moderately differentiated adenocarcinoma; 5 = poorly differentiated adenocarcinoma or small cell carcinoma. Prostates containing regions that differed morphologically were assigned a grade reflecting the most prevalent region. Tumors were also graded according to the extent of involvement: 1 = focal; 2 = multi-focal; 3 = diffuse. Tumor score was calculated as tumor grade × tumor extent.

### Efficacy studies in metastatic models

To model prostate cancer metastatic to the liver, tumor cells were injected into the hemi-spleens of ProHA × TRAMP mice or B10.D2 mice using a previously described surgical procedure [33]. Briefly, the spleens of anesthetized mice were divided into halves and each half individually clipped. SP1 cells (1.0 × 10^5^) were injected into one hemispleen. After 30 seconds, the injected hemispleen was resected and the corresponding splenic vein was clipped. For the pulmonary metastasis model, tail vein injection of 1.0 × 10^5^ SP1 cells suspended in 200 μl HBSS using a 26-guage needle was performed. GVAX immunotherapy using SP1 cells (see above) was administered 3 days after tumor injection and anti-CTLA-4 mAb was injected per the indicated schedule. Cyclophosphamide (50 mg/kg) was administered 1 day before GVAX.

### Statistical analyses

Unless otherwise indicated, each experiment was performed in triplicate using a minimum of 5 animals per group. Representative results are shown. Mean ± SEM is shown. For comparisons between groups, a one-way ANOVA with post-test comparison was performed. A log-rank test was performed for survival. Differences were considered statistically significant for two-sided *p* values < 0.05. Calculations were performed using the GraphPad PRISM package (GraphPad, La Jolla, CA).

## Results

### Combination therapy with anti-CTLA-4 monoclonal antibody and GVAX immunotherapy promotes proliferation of tumor antigen-specific CD8 T cells

**Figure 1:**
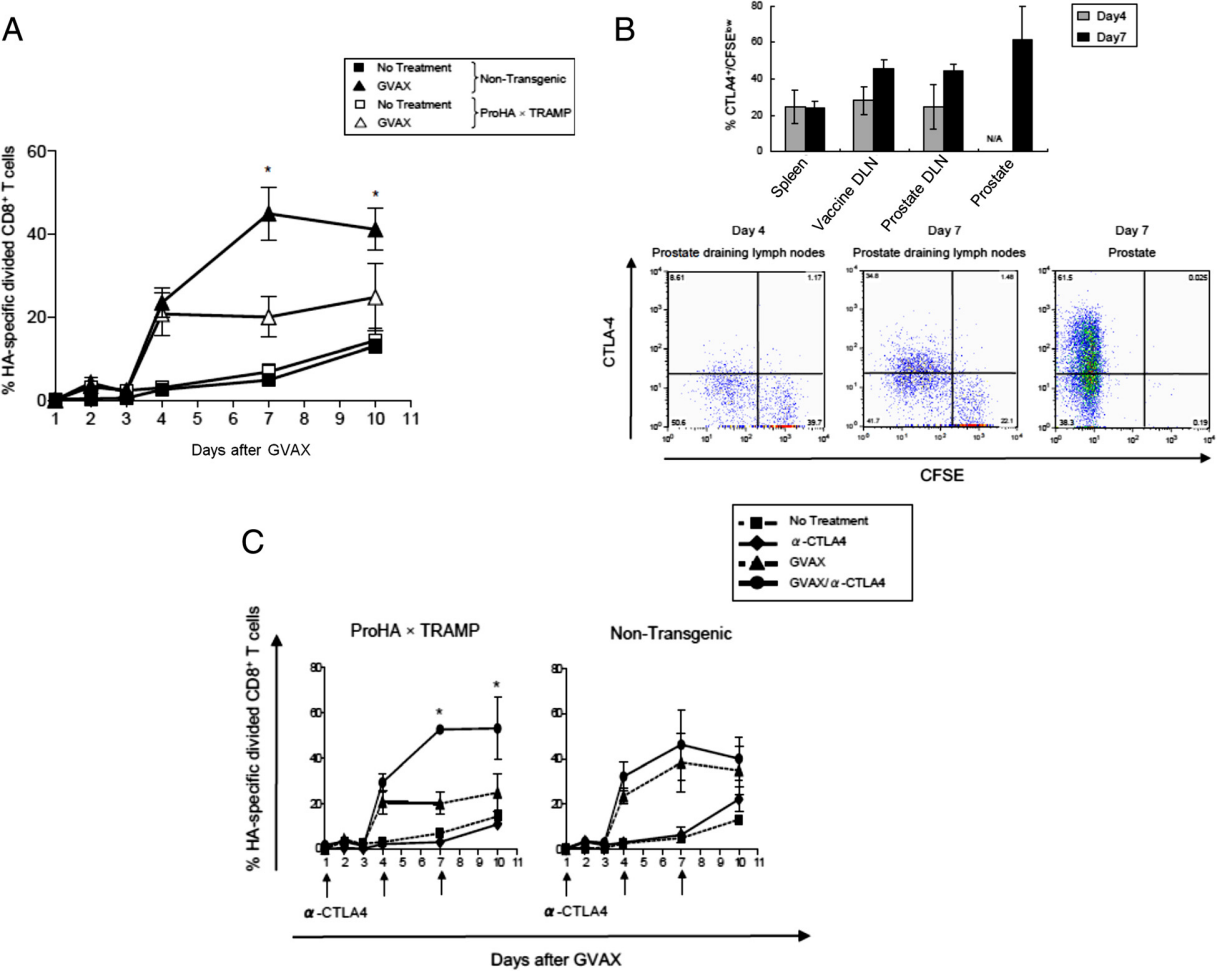
**GVAX Immunotherapy induces CTLA-4 expression on tumor-specific CD8 T cells. A**: Blunted CD8 T cell response to GVAX immunotherapy in tumor-bearing mice. CFSE-labeled HA-specific CD8 T Cells (Clone 4) were adoptively transferred to non-transgenic mice (B10.D2) or tumor bearing mice (ProHA x TRAMP), and allowed to equilibrate in vivo for 2 days prior to treatment with GVAX immunotherapy (Day 0). Peripheral blood cells were harvested by tail vein aspiration on the indicated days post-immunization. Data shown are gated on HA-specific CD8^+^ Thy1.1^+^ lymphocytes. Mean ± SEM shown, 5 animals per group, representative of 2 experiments. *P < 0.05 (GVAX treated ProHA x TRAMP vs. Non-Transgenic). **B**: CTLA-4 expression in tumor-specific CD8 T Cells. Top panel: indicated organs were harvested on day 4 and day 7 post-immunization. Data shown are gated on HA-specific CD8^+^ Thy1.1^+^ lymphocytes that divided at least once. CTLA-4 expression was determined by intracellular staining. Mean ± SEM shown, 3 animals per group, representative of 2 experiments. Bottom panel: representative dot plots for CFSE and CTLA-4 staining, gated on HA-specific (CD8^+^ Thy1.1^+^) T cells. **C**: Effects of CTLA-4 blockade on expansion of HA-specific CD8^+^ T Cells. CFSE-labeled HA-specific CD8^+^ T Cells were adoptively transferred to indicated mice and animals were treated 2 days post-transfer with GVAX immunotherapy (Day 0). Anti-CTLA-4 was administered on the indicated days. Peripheral blood cells were harvested on indicated days. Data shown are gated on HA-specific CD8^+^ Thy1.1^+^ lymphocytes. Mean ± SEM shown, 5 animals per group, representative of 2 experiments.*P < 0.05 (GVAX/anti-CTLA-4 vs. GVAX alone).

To evaluate the systemic immunologic effects of cell-based immunotherapy (GVAX) in either non-transgenic or tumor-bearing ProHA × TRAMP mice, we adoptively transferred CFSE-labeled, HA-specific CD8 T cells. The proliferation and effector function of these cells reflects the relative efficacy of vaccination in the respective strains. Proliferation of HA-specific CD8 T cells (which represent prostate/prostate-cancer specific T cells in this model) was assessed by dilution of CFSE. As shown in Figure 1A, GVAX immunotherapy resulted in a robust increase in the percentage of divided CD8 T cells specific for HA in both tumor-bearing and non-transgenic recipients. Division peaked approximately 7 days post-vaccine administration in non-transgenic mice, but plateaued on day 4 in tumor bearing mice. As is typical for naïve CD8 T cells, some background proliferation was detectable in non-transgenic animals. To determine whether expression of the immune checkpoint molecule CTLA-4 could potentially be restraining the vaccine response of prostate/prostate cancer specific cells, we stained the prostate-specific CD8 T cells for CTLA-4 expression. As shown in Figure 1B, CTLA-4 expression in the prostate-draining lymph nodes was noted on day 4, with approximately 16% of divided cells staining positive. By day 7, expression was more robust; at this time point approximately 45% of the HA-specific CD8 cells in the draining lymph node expressed CTLA-4. In the tumor-bearing prostate gland itself, more than half of the specific CD8 T cells expressed CTLA-4. Taken together, these data show that GVAX vaccination induces a detectable, but relatively blunted CD8 T cell response in prostate tumor-bearing mice, and this blunted response correlates with CTLA-4 expression on specific T cells, especially in the target organ.

Based on these data, we evaluated the ability of a CTLA-4 blocking monoclonal antibody to augment vaccine-mediated proliferation, once again using either non-transgenic, or tumor bearing mice (ProHA × TRAMP). Animals received infusions of CFSE-labeled HA-specific CD8 T cells as readout for vaccine (+/− antibody) efficacy. As expected, anti-CTLA-4 treatment alone did not significantly increase the percentage of divided HA-specific CD8 T cells in either tumor bearing or non-tumor bearing mice (Figure 1C). In tumor bearing mice, combination therapy significantly increased the percentage of HA-specific CD8 T cells as compared with GVAX treatment alone, while in non-tumor bearing mice, the addition of anti-CTLA-4 mAb did not significantly increase the percentage of HA-specific T cells beyond the effect observed with GVAX alone. These data support the concept of GVAX + anti-CTLA-4 combination therapy, and suggest that the effects of combination treatment may be more pronounced in the presence of cancer.

### Timing and dosage of anti-CTLA-4 antibody is critical for augmenting GVAX immunotherapy

**Figure 2:**
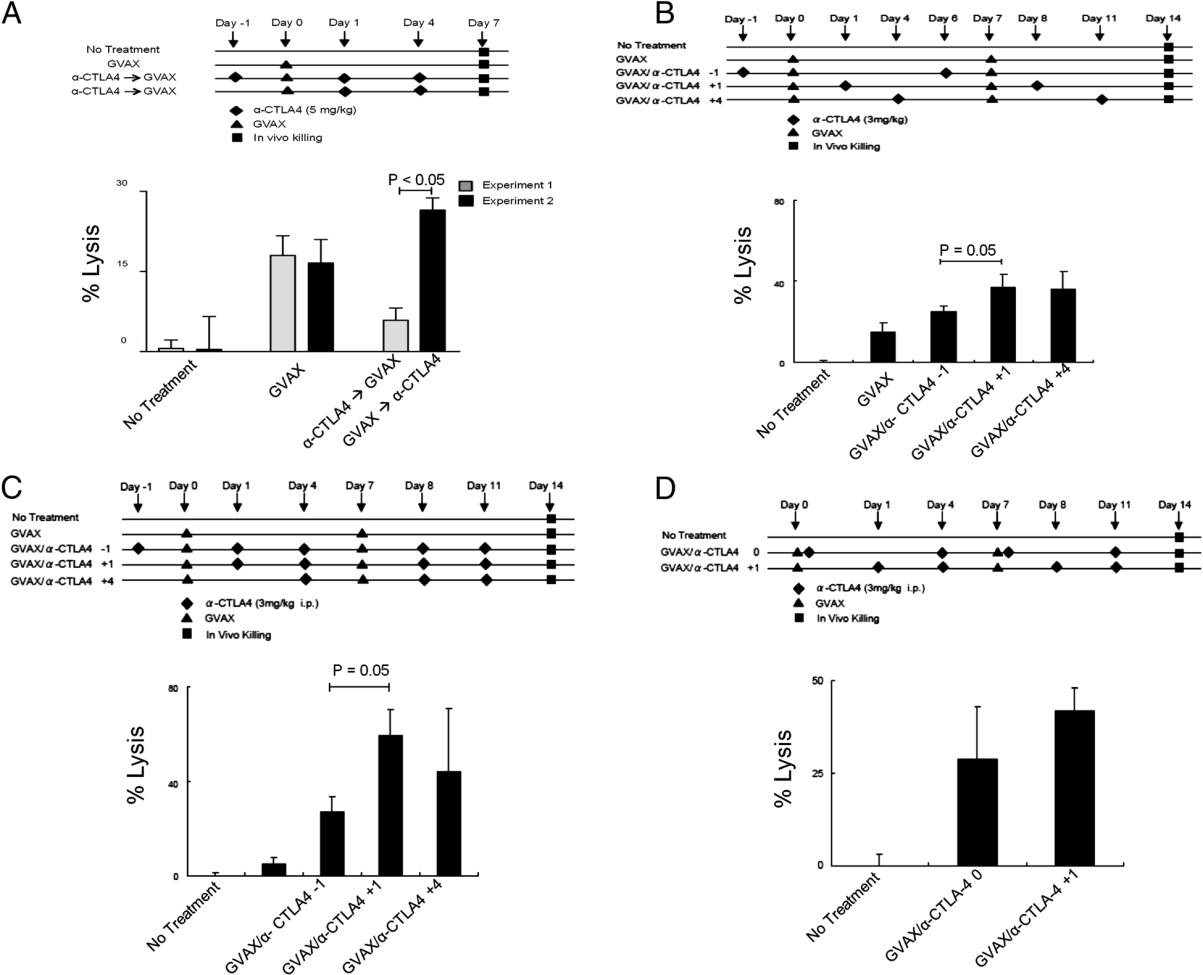
**Time-dependent augmentation of anti-tumor CD8 T cell response by the addition of CTLA-4 blockade to GVAX immunotherapy. A**: Timing of GVAX immunotherapy and CTLA-4 blockade in non-transgenic mice. Top panel shows experimental design. Readout was HA-specific lytic function quantified by an *in vivo* CTL analysis. Endogenous T cell lytic function was assayed; no adoptive T cell transfer was performed. 5 animals per group, representative of 2 experiments. **B**: Timing of GVAX immunotherapy and CTLA-4 blockade in tumor-bearing, ProHA x TRAMP mice. Top panel: experimental design. Bottom panel: CTL function in ProHA x TRAMP mice. As in Figure 2A these studies were performed without adoptive T cell transfer, i.e. endogenous T cell lytic activity was assayed. 5 animals per group, representative of 2 experiments. **C**: Effects of more frequent anti-CTLA-4 administration. Experimental design (top panel) is identical to Figure 2B, with the exception of more frequent anti-CTLA-4 administration. 5 animals per group, representative of 2 experiments. **D**: Day 0 vs. Day 1 anti-CTLA4 dosing. Experimental design (top panel). Dosing of anti-CTLA-4 on Day 1 was directly compared with same day (Day 0) GVAX/anti-CTLA-4 dosing. 5 animals per group, representative of 2 experiments.

The treatment schema used in Figure 1C was empiric; we attempted to relatively saturate CTLA-4 with antibody by treating with three doses 2 days apart. To more directly examine the immunological effects of combination treatment in tumor-bearing mice, we performed a series of studies varying the relative timing of vaccination and CTLA-4 blockade, in either non-transgenic or tumor-bearing ProHA × TRAMP mice. We also used a more functional readout for efficacy, *in vivo* lysis of HA-loaded target cells by the endogenous (as opposed to adoptively transferred) CD8 T cell population. In tumor-free, non-transgenic mice, GVAX vaccination alone resulted in a significant CTL response detectable 7 days after vaccination (Figure 2A). Administration of 2 doses (5 mg/kg) of anti-CTLA-4 after vaccination increased the relative CTL response as compared to GVAX vaccination alone. Interestingly, in non-transgenic mice, the opposite sequence was not successful. Administration of anti-CTLA-4 before vaccination did not increase the specific CTL response and, in fact, the response appeared to be slightly diminished. In non-transgenic mice, the difference between these two sequences was statistically significant, with checkpoint blockade clearly demonstrating greater efficacy when given after vaccination.

We next performed similar studies in tumor-bearing ProHA × TRAMP mice (Figure 2B). Since multiple previous studies by our group have shown that a single dose of GVAX in tumor-bearing mice does not induce a detectable lytic response [23], we gave two doses of vaccine, spaced one week apart, in this study. As in non-transgenic mice, the lytic response in tumor-bearing mice was optimal when anti-CTLA-4 was administered after vaccination. Interestingly, giving anti-CTLA-4 several days post vaccination still augmented the CTL response, as might be suggested by the kinetics of CTLA-4 expression shown in Figure 1. We also evaluated a more extensive anti-CTLA-4 treatment regimen in which three doses of anti-CTL-4 were administered with each GVAX vaccine dose. As shown in Figure 2C, optimal timing of vaccination and checkpoint blockade was not affected by this more intensive treatment protocol; once again maximum lytic function was observed with anti-CTLA-4 administration one day post vaccination. Due to the expected convenience of same-day dosing in a clinical setting, we also examined whether anti-CTLA-4 blockade could be given at the same time as GVAX vaccination. We found no significant difference between administering anti-CTLA-4 on day 1 vs. day 0 (Figure 2D). Taken together, these data support a combination regimen in which anti-CTLA-4 is administered on the day of or following GVAX immunotherapy.

**Figure 3:**
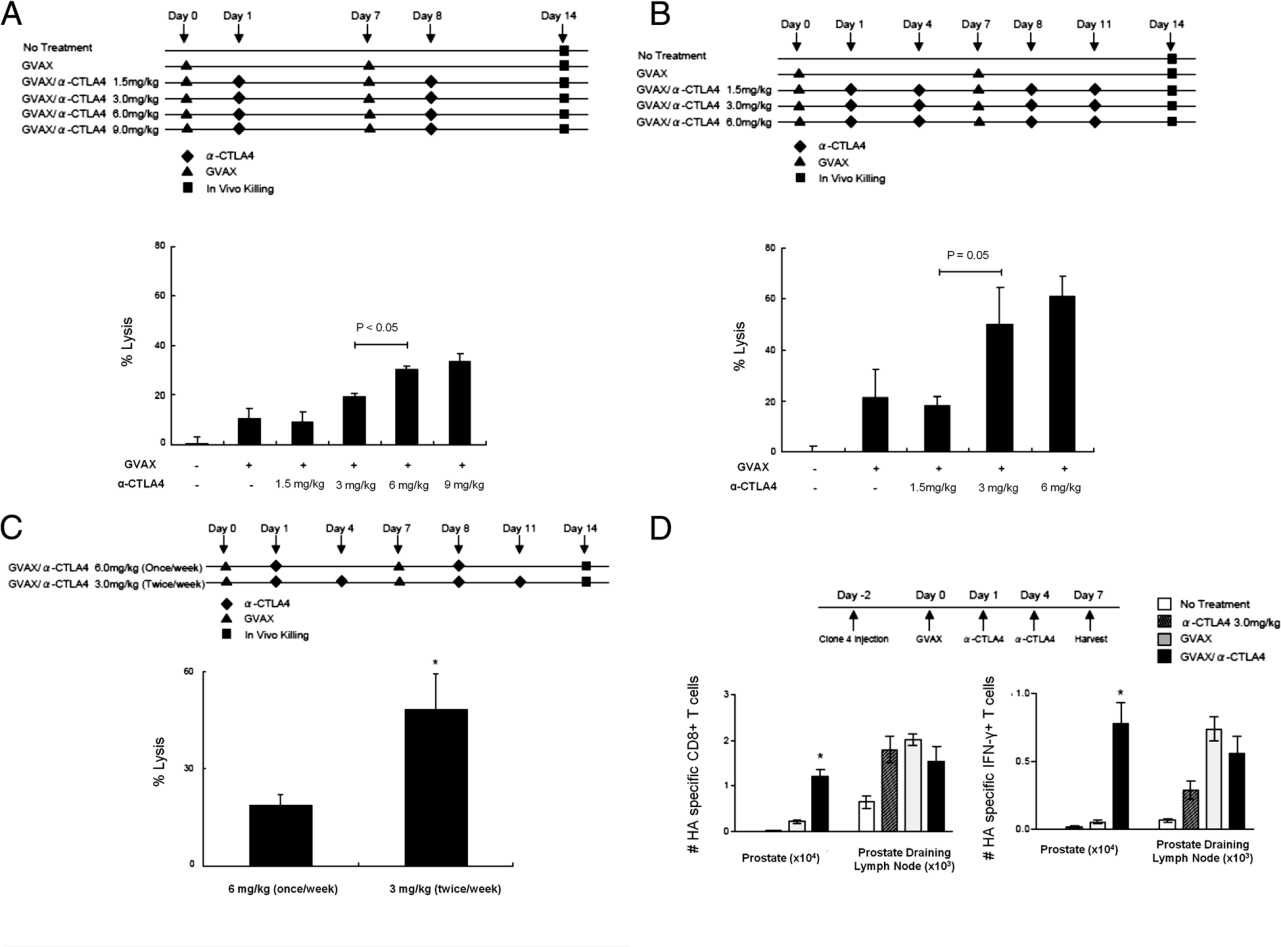
**Optimization of anti-CTLA-4 dosing in combination with GVAX immunotherapy. A**: Titration of anti-CTLA-4 using once per week dosing. Top panel: experimental design. Bottom panel: CTL function in ProHA x TRAMP mice. 5 animals per group, representative of 2 experiments. **B**: Titration of anti-CTLA-4 using twice per week dosing. Top panel: experimental design. Bottom panel: CTL function in ProHA x TRAMP mice. 5 animals per group, representative of 2 experiments. **C**: Comparison of single versus divided dosing of anti-CTLA-4 per week. Top panel: experimental design. Bottom panel: CTL function in ProHA x TRAMP mice. 5 animals per group, representative of 2 experiments.*P < 0.05. **D**: Prostate directed expansion of specific CD8 cells: Top panel: experimental schema. Bottom panels: Absolute numbers of CD8 T cells harvested from prostate or prostate draining lymph nodes. Left: Absolute number of IFN-γ positive (by intracellular staining) CD8 T cells in those locations. 3 animals per group, representative of 2 experiments. *P < 0.01 (GVAX/α-CTLA-4 mAb vs. GVAX alone).

As clinical data suggest that the development of IRAEs is dose-dependent (http://www.fda.gov), we next sought to determine the minimal dose of anti-CTLA-4 mAb required to achieve additive effects. In these studies, we once again tested the effects of a single antibody treatment per vaccine (Figure 3A) as well as a more dose dense regimen (Figure 3B). We found that a dose of 6 mg/kg in a once-per-week dosing paradigm was significantly more effective than 3 mg/kg or 1.5 mg/kg in promoting endogenous CTL function. Although the data approximate a dose–response relationship, the difference in CTL function between 6 mg/kg and 9 mg/kg dosing was not significant. In a dose-dense regimen, we found that an intraperitoneal dose of 3 mg/kg was significantly more effective than 1.5 mg/kg and there was no significant difference between 3 mg/kg and 6 mg/kg (Figure 3B). After finding that 6 mg/kg/week produced a maximal CTL response, we directly compared once per week and twice per week dosing regimens at this dose (Figure 3C). A dose of 3 mg/kg (twice/week) resulted in increased lytic function compared with the same quantity of antibody administered in a single weekly bolus at 6 mg/kg. Taken together these data suggest that relatively low-dose exposure to CTLA-4 blockade might be more efficacious than less frequent high-dose boluses.

We next returned to an adoptive transfer model to ascertain whether this regimen would promote expansion and/or effector function of prostate/prostate cancer specific CD8 T cells. As shown in Figure 3D, the effects of the combination regimen were most notable in the target organ, where a several fold expansion in the absolute number (left panel) of HA-specific CD8 T cells was noted. In addition, the majority of these cells secreted interferon-gamma (IFN-γ) (right panel), consistent with the acquisition of effector function. These data provide a mechanistic basis for the increased lytic function mediated by the combination regimen.

### Combination immunotherapy with CTLA-4 blockade and GVAX decreases tumor burden and histologic grade in ProHA x TRAMP mice

**Figure 4:**
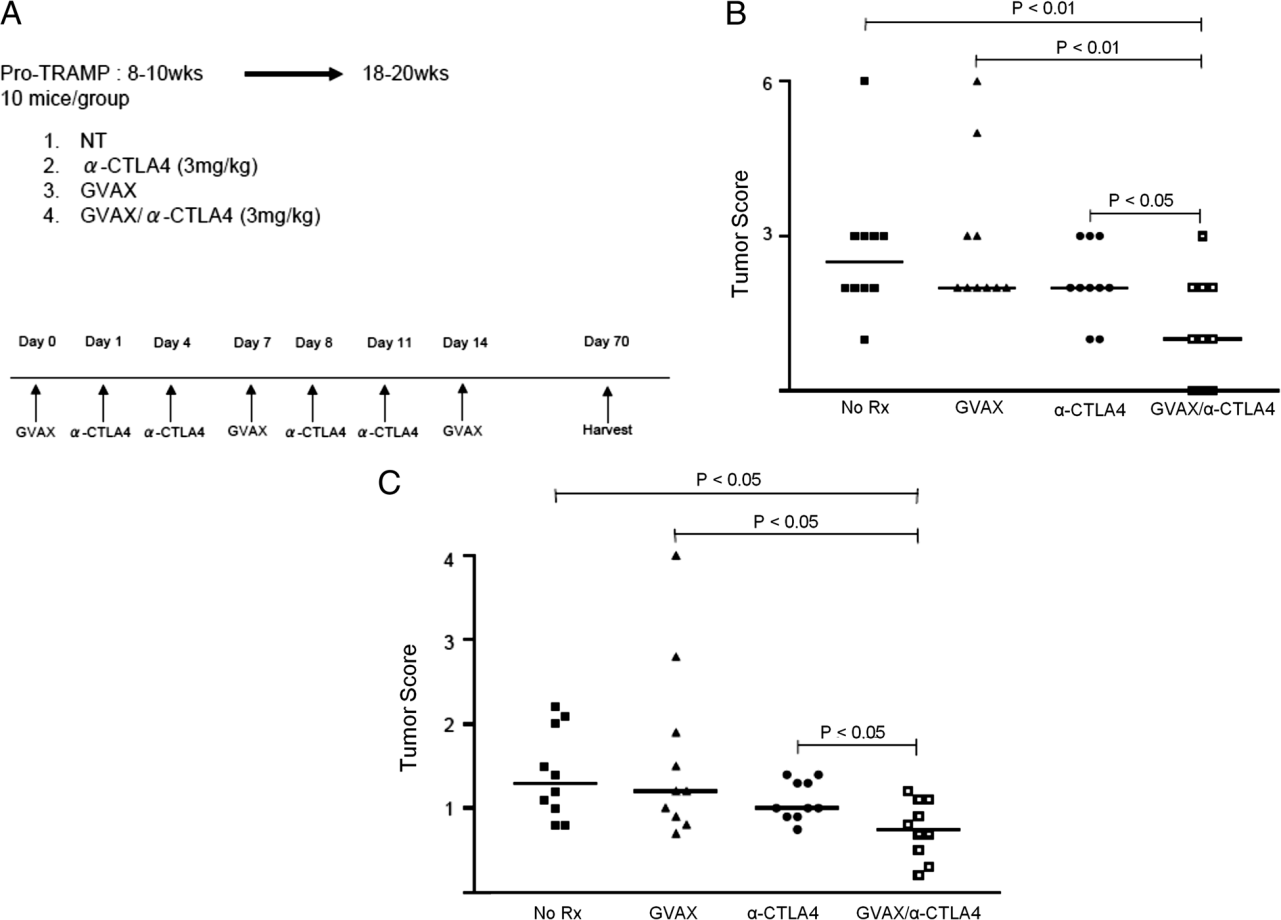
**Longitudinal treatment studies in mice bearing autochronous prostate tumors. A**: Experimental design. **B**: Tumor scores. Tumor score was calculated by multiplying pathological score x extent. All samples were evaluated by two independent pathologists blinded as to treatment group assignment (See Material and methods). **C**: Wet weights of the urogenital tract. N = 10 animals per group.

To assess whether anti-CTLA-4 could mediate an additive or synergistic anti-tumor effect, we performed longitudinal treatment studies using ProHA × TRAMP mice. Age-matched 8–10 week old animals (corresponding to early disease), were randomly assigned to one of 3 treatment arms: GVAX, anti-CTLA-4, or GVAX + anti-CTLA-4 mAb. A schematic of the treatment regimen is depicted in Figure 4A. Ten weeks after initial treatment, animals were sacrificed and tumor grade and extent were evaluated in a blinded manner as previously described [21,23]. As shown in Figure 4B, scoring of the microdissected ventral lobes of the prostate glands revealed a significant decrease in tumor score with combination treatment as compared with either treatment alone. Both GVAX and anti-CTLA-4 monotherapy demonstrated a non-significant trend toward efficacy as compared with untreated control animals. The wet weight of the urogenital tract, a gross surrogate for tumor burden [34], corroborated these data nicely (Figure 4C). Taken together, these data support a treatment regimen in which relatively low doses of anti-CTLA-4 are given post-vaccine.

### The combination of GVAX and CTLA-4 blockade alters the phenotype of prostate/prostate-tumor specific CD4 T cells

**Figure 5:**
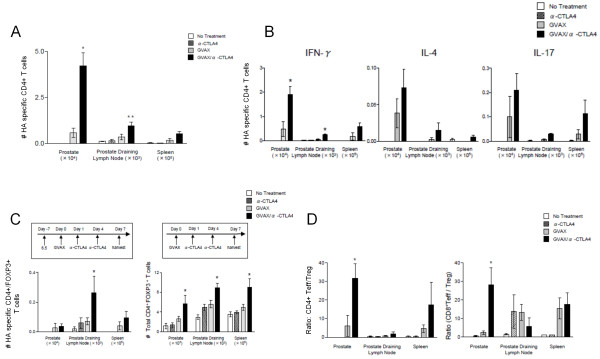
**Tumor-specific CD4**^**+ **^**T cell subsets induced by combined therapy. A**: Prostate-directed accumulation of HA-specific CD4^+^ T cells. Prostate/prostate-cancer specific CD4 T cells from transgenic mice (6.5 strain) were adoptively transferred to tumor-bearing ProHA x TRAMP mice. After 2 days of *in vivo* equilibration, mice were treated with GVAX immunotherapy, followed by two doses of anti-CTLA-4 (3 mg/kg) as shown in the top left panel of Figure 5D. Clonotypic (prostate-specific) CD4 were selected by staining for CD4 and the congenic marker Thy1.1. N = 3 animals per group, representative of 2 experiments. *P < 0.01 (GVAX/anti-CTLA-4 vs GVAX alone), **, P < 0.05 (GVAX/anti-CTLA-4 vs GVAX alone). **B**: CD4 T cell subsets expanded by a combination treatment regimen. HA-specific CD4^+^ T cells were evaluated for secretion of the indicated cytokines by intracellular staining after a brief ex-vivo stimulation. N = 3 animals per group, representative of 2 experiments. *P < 0.05 (GVAX/anti-CTLA-4 vs. GVAX alone). **C**: Combination treatment expands both specific and endogenous regulatory T cells (Treg). Top panel: experimental design. Bottom Left: HA-specific CD4^+^FoxP3^+^ T cells quantified using intracellular staining of lymphocytes harvested from the indicated organs. Bottom right: Endogenous Treg (experiments performed without adoptive transfer). N = 3 animals per group, representative of 2 experiments.*P < 0.05 (GVAX/anti-CTLA-4 mAb vs GVAX alone). **D**: Effects of combination treatment on the Effector / Treg ratio. Prostate / prostate-cancer specific CD4^+^ and CD8^+^ effector T cells (IFN-γ positive) were quantified on day 7 following administration of GVAX + anti-CTLA-4 combination therapy, and ratios determined by dividing by numbers of Treg as quantified using FoxP3^+^ intracellular staining. N = 3 animals per group, representative of 2 experiments.

We next sought to evaluate the impact of anti-CTLA-4 and GVAX combination immunotherapy on tumor antigen-specific CD4 T cell numbers and function. Similar to our prior studies of CD8 cells, HA-specific CD4 T cells (6.5) were adoptively transferred into ProHA × TRAMP mice 7 days prior to GVAX treatment. As shown in Figure 5A, in the absence of specific vaccination, these cells are difficult to detect after one week in tumor-bearing mice. This relative lack of persistence was not affected by anti-CTLA-4 treatment to any appreciable degree; however, GVAX vaccination resulted in a detectable population of specific CD4 T cells within the prostate gland and prostate draining lymph nodes (Figure 5A). Notably, the combination of anti-CTLA-4 and GVAX vaccination significantly increased the number of tumor infiltrating CD4 T cells in the prostate gland, as compared to GVAX therapy alone (Figure 5A). We next performed intracellular staining (after a brief ex-vivo stimulation) to determine the phenotype of the specific CD4 T cells. As shown in Figure 5B, combination immunotherapy in ProHA × TRAMP mice resulted in a significant increase in anti-tumor T_H_1 CD4 cells within the local tumor environment and in the draining lymph nodes (Figure 5B). In contrast, although there was a trend toward increased numbers of IL-4 and IL-17 producing tumor antigen-specific CD4 T cells both systemically and locally, these trends were not statistically significant.

### CTLA-4 and GVAX combination therapy promotes expansion of Tregs, but increases antigen-specific CD4+ and CD8+ Teff/Treg ratios

As Tregs have been demonstrated to restrain an anti-tumor immune response [35,36], we next examined the impact of combination immunotherapy on the number of tumor antigen-specific and total Tregs. GVAX + anti-CTLA-4 mAb combination therapy did not reduce the number of tumor antigen-specific Tregs (Figure 5C). In fact, therapy resulted in a significant increase in the absolute numbers of HA-specific CD4^+^/Foxp3^+^ T cells in prostate draining lymph nodes. A similar pattern was observed for total numbers of CD4^+^/Foxp3^+^ T cells, both systemically and locally. Prior studies have demonstrated that while combined therapy with GVAX and CTLA-4 blockade may result in increased absolute numbers of Tregs, increases in the Teff:Treg ratio are characteristic of an effective anti-tumor response [37]. Hence, we evaluated the effects of CTLA-4 blockade and GVAX combination therapy on the tumor antigen-specific Teff and Treg populations within the tumor and tumor-draining lymph nodes in our ProHA × TRAMP model. As shown in Figure 5D, CTLA-4 blockade and GVAX treatment resulted in an increase in the ratio of tumor antigen-specific CD4 and CD8 Teffs to Tregs. Interestingly, this effect was quite dramatic in tumor tissue, for both CD4 and CD8 T cells. Taken together these data support a model wherein combination treatment increases the number of both CD4 and CD8 effector cells, in addition to a relative increase in the Teff: Treg ratio for both T cell subtypes.

### Transient depletion of tregs with low-dose cyclophosphamide augments the anti-tumor effects of combination immunotherapy

**Figure 6:**
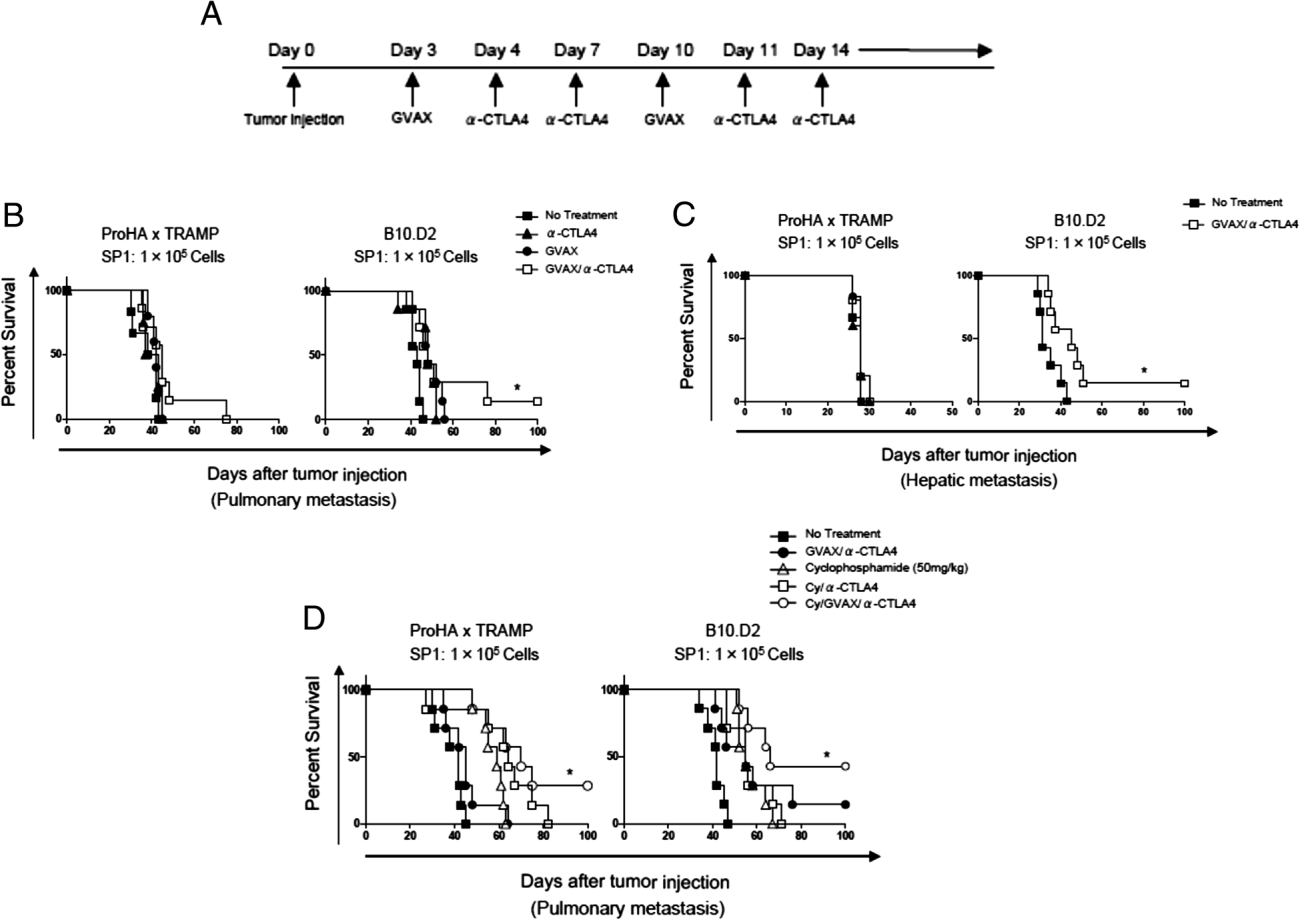
**Anti-tumor effects of combined therapy in models of pulmonary and hepatic metastases. A**: Treatment regimen. **B**: Treatment effect in a pulmonary metastasis model. Cohorts of 20–22 week old ProHA x TRAMP mice (left panel) or non-transgenic (right panel) received I.V. injections of tumor cells (SP1) and were then treated with either combination or single-agent immunotherapy. N = 7 animals per group, representative of 2 independent experiments. *P < 0.05 (GVAX/anti-CTLA-4 vs. No Treatment.). **C**: Treatment effect in a hepatic metastasis model. Cohorts of 20–22 week old ProHA x TRAMP mice (left panel) or non-transgenic (right panel) received intra-splenic injection of tumor cells (SP1), followed by resection of the injected hemispleen to model isolated heptatic metastases. Animals were then treated with either combination immunotherapy or left untreated. N = 7 animals per group, representative of 2 independent experiments. *P < 0.05 (GVAX/anti-CTLA-4 vs No Treatment.). **D**: Addition of low-dose cyclophosphamide to combination immunotherapy. 20–22 week ProHA x TRAMP mice or non-transgenic mice were intravenously injected with the prostate cancer cell line (SP1). Cyclophosphamide (50 mg/kg) was administered 1 day before each GVAX injection. The treatment regimen was otherwise identical to that shown in 6A. N = 7 animals per group. Representative of 2 experiments. *P < 0.05 (Cy/GVAX/anti-CTLA-4 vs GVAX/anti-CTLA-4).

Although multiple studies have shown that tumor immunotherapy is most likely to be successful in a setting of lower disease burden [38], the majority of clinical studies have been conducted in men with castrate-resistant, metastatic disease [8]. We thus sought to determine the relative efficacy of combination GVAX/anti-CTLA-4 therapy in models of metastatic disease, both in the presence and absence of primary tumor. For these studies, we derived a unique cell line (SP1) from the prostate gland of a late-stage ProHA × TRAMP mouse. This line, SP1, is class I positive, and expresses low levels of HA. We first modeled pulmonary metastases (by I.V. injection of tumor cells) in both wild type and tumor bearing mice. Three days after tumor cell injection, mice were treated with either combination therapy (GVAX + anti-CTLA-4), or each treatment alone (Figure 6A). As show in Figure 6B, combination treatment was not able to significantly delay mortality on the tolerant ProHA × TRAMP background. Similar results were noted in non-tolerant B10.D2 (wildtype mice), but here a small proportion of mice were able to survive long-term. These data underscore the challenges involved in treating metastatic disease, particularly in the setting of pre-existing tolerance. Similar results were obtained in a model of liver metastases [33] as a significant survival benefit was observed only in non-transgenic mice (Figure 6C).

In a final series of studies, we explored the possibility that the expansion of Tregs mediated by GVAX and anti-CTLA-4 combination treatment limits the immune response. We thus repeated the pulmonary metastasis studies shown in Figure 6B, with the addition of low dose cyclophosphamide (50 mg/kg) to our treatment regimen [23]. As shown in Figure 6D, the addition of cyclophosphamide significantly improved overall survival in animals with concurrent primary and metastatic disease as well as non-transgenic animals with metastatic tumors, reliably resulting in a small population of long-term disease-free mice in endogenously tolerant (ProHA × TRAMP) as well as wild-type mice.

## Discussion

Ipilimumab, a monoclonal antibody blocking the immune checkpoint CTLA-4, has recently been evaluated in two randomized, Phase III, placebo-controlled trials in men with metastatic, castrate-resistant prostate cancer. In one study, the agent was administered as monotherapy to chemotherapy-naïve men (NCT01057810). In the second trial (NCT00861614), ipilimumab was administered in combination with low-dose radiotherapy to one or more bone lesions in patients who had progressed after docetaxel chemotherapy. Mature data from these trials are not yet available. Combination regimens involving ipilimumab and immunotherapy have also been evaluated in Phase I clinical trials. Recently, Madan et al. showed that ipilimumab could be safely combined with the PSA-targeted vaccine PSA-Tricom, without significantly exacerbating the agent’s immune-related adverse event profile [39]. Interestingly, over 50% of the patients in this study who were chemotherapy-naïve experienced a PSA decline from baseline levels. Perhaps most relevant to our current data, the Gerritsen group performed a Phase I study to determine whether ipilimumab could safely be combined with GVAX prostate [40]. Those results were also encouraging, demonstrating the tolerability of the combined regimen and providing early evidence for efficacy. Taken together, these clinical trials highlight a need for preclinical studies that use relevant (immunologically tolerant) animal models to optimize dosing and sequencing.

We examined the anti-tumor effects of combination therapy with GVAX and anti-CTLA-4 in an autochthonous prostate cancer model expressing HA in a prostate-restricted manner. Although previous preclinical studies have demonstrated that CTLA-4 blockade augments the anti-tumor effects of GVAX [18], to our knowledge this is the first study to report extensively on the importance of timing and dosage in this treatment regimen. Our data show that GVAX + anti-CTLA-4 combination therapy mitigates peripheral tolerance and promotes the activation and proliferation of tumor antigen-specific CD8 T cells. While GVAX immunotherapy alone induced proliferation of HA-specific CD8 T cells in non-transgenic animals, this effect was significantly blunted in ProHA × TRAMP mice, confirming previous findings that T cells become refractory to tumor antigens in the setting of endogenously arising prostate tumors [19]. In addition, our results indicate that the percentage of T cells comprising the CD8 HA-specific T cell population increased up to day 7 post vaccination in non-transgenic animals, but reached a plateau at day 4 in tumor-bearing mice. Expression of CTLA-4 on CD8 HA-specific T cells in prostate and prostate draining lymph nodes significantly increased between days 4 and 7 in tumor bearing mice. Consistent with previous studies [20], these data indicate that ProHA × TRAMP mice harbor tumors which induce tolerance that is mediated, at least to some degree, by increased CTLA-4 expression on CD8 T cells, although effects on CD4 T cells, as well as priming stages of the immune response, are likely also involved.

Administration of GVAX followed by anti-CTLA-4 resulted in a significant increase in the percentage of HA-specific CD8^+^ T cells at days 7 and 10. The efficacy of this treatment regimen, however, was highly dependent upon the dose and relative timing of GVAX and anti-CTLA-4 administration. Interestingly, while administration of anti-CTLA-4 at days 1 and 4 post-GVAX resulted in a significant increase in target cell lysis, this effect was not observed when anti-CTLA-4 was administered on day −1. There are a number of potential explanations for this effect. Perhaps the simplest explanation is that the anti-CTLA-4 mAb migrates to multiple sites of CTLA-4 expression (i.e. the gut), and that expression of CTLA-4 on tumor-specific T cells is not particularly robust until after antigen-specific vaccination. This explanation is supported by our data, which show relatively delayed kinetics of CTLA-4 expression after vaccination (Figure 1). A second possibility is that the administration of anti-CTLA-4 results in a compensatory expansion of the Treg compartment [14] and thus prevents generation of an effective anti-tumor response when GVAX vaccine is administered later. While additional studies might be helpful in elucidating the underlying mechanism, it is clear from our data that administration of GVAX prior to anti-CTLA-4 was superior in stimulating anti-tumor CD8 T cell activity as compared with upfront administration of anti-CTLA-4 or either therapy alone. This finding has clear clinical significance. In addition, our data demonstrate that there was no difference in administration of anti-CTLA-4 mAb on day +1 vs. day +4 in once-per-week or twice-per-week dosing regimens. Anti-CTLA-4 mAb dose-escalation studies demonstrated the greatest effects between 3 mg/kg and 6 mg/kg in the once per week dosing regimen and, correspondingly, 1.5 mg/kg and 3 mg/kg in the twice per week dosing regimen. In a direct comparison of dosing regimens at 6 mg/kg/week, we found twice per week dosing to be superior to once-per-week dosing. Based on these data, we concluded that the treatment regimen that produced optimal CD8 T cell anti-tumor activity was day 0 GVAX followed by twice per week anti-CTLA-4 mAb at the dose of 3 mg/kg.

After determining the optimal treatment regimen for GVAX + anti-CTLA-4 in our ProHA × TRAMP model, we sought to more thoroughly explore the underlying immunologic mechanisms. Our data clearly demonstrate an increase in the absolute number of HA-specific CD8 T cells as well as an increase in the number of IFN-γ producing cells in the prostate gland and prostate draining lymph nodes. Interestingly, there was also a trend toward decreased numbers of HA-specific CD8 T cells in the prostate draining lymph nodes with combination therapy as compared with GVAX therapy alone. Given that this effect was not observed in prostate tissue, it is reasonable to speculate that treatment with anti-CTLA-4 mAb may increase trafficking of tumor antigen-specific T cells into tumor tissue [41].

Although combination therapy with GVAX and CTLA-4 blockade increases tumor antigen-specific CD8 T cell proliferation and activation, this has not been consistently shown to correspond with tumor eradication or improved survival [42], so we used tumor score and weight of the genital tract as direct measures of effectiveness in a relevant preclinical model. We found that the tumor score was significantly lower for animals receiving combination therapy as compared with control animals or animals receiving either therapy alone. Of note, 3 of the 10 mice receiving combination therapy had no histologic evidence of disease, while tumors were identified in every mouse in the other treatment arms. The weight of the urogenital tract corroborated these findings as the urogenital tracts of animals receiving combination therapy weighted significantly less than animals in the other treatment arms. These data support the potential clinical utility of the combination regimen.

In addition to expanding the CD8 T cell compartment, CTLA-4 blockade has been shown to affect various CD4 T cell populations, including Tregs [43] and Th17 cells [44]. Therefore, we evaluated the response of these cell populations to our treatment regimen. Combination therapy resulted in skewed local and systemic expansion of the HA-specific CD4 T cell compartment toward an anti-tumor, IFN- γ^+^ phenotype. Our data further suggest that this expansion may include other CD4 T cell subtypes as there was a non-statistically significant trend toward increased numbers of HA-specific CD4^+^/IL-4^+^, CD4^+^/IL-17^+^, and CD4^+^/FoxP3^+^ T cells. Expansion of the Th17 compartment is consistent with previous studies [44] and, although these cells have been traditionally considered pro-tumorigenic, there is some evidence to suggest that they may play a role in anti-tumor immune responses as well [45]. The finding that the numbers of HA-specific and total Tregs are increased is also consistent with previous studies [14]. Our data suggest that, while combination treatment induces Treg expansion, treatment also increases tumor antigen-specific CD4 Teff/Treg and CD8 Teff/Treg ratios.

Since depletion of Tregs may be associated with improved survival in patients with metastatic castration-resistant prostate cancer responding to vaccines [46], we sought to determine whether the addition of low-dose cyclophosphamide further augments the GVAX + anti-CTLA-4 treatment effect in models of pulmonary and hepatic metastasis. Analogous to our previous finding that GVAX alone failed to induce HA-specific CD8 T cell proliferation in ProHA × TRAMP mice, combination therapy increased survival in non-transgenic animals with pulmonary or hepatic metastases, but failed to do so in ProHA × TRAMP animals harboring autochronous prostate tumors. The addition of cyclophosphamide (50 mg/kg) to this treatment regimen, however, resulted in improved survival in the non-transgenic animals and rescued the survival advantage in ProHA × TRAMP animals. Mechanistically, these results generally support the notion that the Teff/Treg ratio correlates with anti-tumor immunity in immunotherapy regimens; however, these data should be interpreted with some caution, since low-dose cyclophosphamide has favorable effects on several other immune cell populations. In particular, this regimen promotes DC secretion of type I IFN, affects DC turnover, and modifies DC phenotype [47]. Thus, there are a number of plausible, competing explanations for the observed effect.

## Conclusion

These experiments corroborate recent clinical data, which suggest that the combination of CTLA-4 blockade and cell-based, GM-CSF-secreting vaccines may have significant anti-tumor effects in men with prostate cancer. Our data indicate that the therapeutic window of such an approach may be maximized through meticulous study of various doses and dosing regimens. Furthermore, Tregs appear to be a limiting factor in this treatment regimen such that depletion of this cell population enhances anti-tumor immunity. Based on these data, clinical studies may find that the addition of cyclophosphamide to this treatment regimen allows for reduction in the dose of anti-CTLA-4, potentially limiting autoimmune toxicity. Since recently reported trials [39,40], show that ipilimumab can safely be administered in combination with immunotherapy to men with prostate cancer, it seems logical to consider the initiation of future trials in which anti-CTLA-4 is combined with GVAX immunotherapy and low-dose cyclophosphamide, most likely in the earlier stages of metastatic disease.

## Competing interests

CGD has consulted for BMS (Princeton NJ) and Pfizer (San Diego, CA), both of whom have developed anti-CTLA-4 antibodies clinically. JFG is currently employed by BMS.

## Authors’ contributions

SW participated in the design and was responsible for carrying out many of the experiments described in this manuscript, analyzing the data, and constructing the figures. CJ participated in data interpretation and writing/revising of the manuscript. KY, HY, DG, TH, MG, TB, JG, ND contributed to experimental design and execution, and also participated in editing and preparing the manuscript. LS and GN were responsible for pathologic grading of prostate tissues. DP participated in data interpretation, experimental design and manuscript revision. CD was responsible for overseeing all experimental design, execution, and data analysis as well as manuscript writing/revision. All authors read and approved the final manuscript.

## Authors’ information

CGD is a Damon Runyon-Lilly Clinical Investigator and is supported by National Institutes of Health R01 CA127153, 1P50CA58236-15, the Patrick C. Walsh Fund, the OneInSix Foundation and the Prostate Cancer Foundation.

Address correspondence and reprint requests to: Dr. Charles G. Drake; Johns Hopkins Sidney Kimmel Comprehensive Cancer Center; 1650 Orleans St, CRB I #410; Baltimore, MD 21231. E-mail address: cdrake@jhmi.edu.
